# Utilization of an Ionic Liquid/Urea Mixture as a Physical Coupling Agent for Agarose/Talc Composite Films

**DOI:** 10.3390/ma6020682

**Published:** 2013-02-22

**Authors:** Ahmad Adlie Shamsuri, Rusli Daik

**Affiliations:** 1 School of Chemical Sciences and Food Technology, Faculty of Science and Technology, Universiti Kebangsaan Malaysia, UKM Bangi 43600, Selangor, Malaysia; E-Mail: adlie@putra.upm.edu.my; 2 Laboratory of Biocomposite Technology, Institute of Tropical Forestry and Forest Products, Universiti Putra Malaysia, UPM Serdang 43400, Selangor, Malaysia

**Keywords:** 1-n-butyl-3-methylimidazolium chloride (BmimCl), polysaccharide, hydrophobic mineral, interfacial adhesion, secondary bonding

## Abstract

An ionic liquid, 1-n-butyl-3-methylimidazolium chloride (BmimCl) was blended with urea at 1:1 mole ratio to create a BmimCl/Urea mixture. The agarose/talc composite films containing the BmimCl/Urea mixture were then acquired through a gelation method. The weight ratio of agarose and talc was fixed at 4:1, while the content of BmimCl/Urea was varied from 0 to 10 wt % relative to the overall weight of the composite films. The tensile stress and modulus results showed the optimum BmimCl/Urea content in the composite film lies at 8 wt %. The talc particles are embedded in the agarose matrix and there are no pullouts for the composite films containing BmimCl/Urea as demonstrated by SEM micrographs. The addition of BmimCl/Urea increased the glass transition temperature of the composite films, however, the thermal decomposition temperature decreased drastically. FTIR and FT-Raman spectra indicated the existence of interaction between agarose and talc, which improves their interfacial adhesion. As a conclusion, a BmimCl/Urea mixture can be utilized as a coupling agent for agarose/talc composite films.

## 1. Introduction

Talc-filled polymer composites exhibit higher mechanical properties (strength and stiffness) than those of the calcium carbonate- and kaolin-filled polymer composites [1]. Incorporation of the talc particles into the polymer has been shown to increase the decomposition temperature as also found for the case of nanoclay addition [2]. The polymer/talc composites produced from polysaccharides are hugely beneficial since these materials are abundant in nature, low-cost, non-toxic and renewable. The composites may also have potential applications in biodegradable, biocompatible and high mechanical characteristic materials. Nonetheless, the interfacial adhesion between the talc particles with polysaccharides in the composites is relatively poor and this ultimately deteriorates the mechanical and other related properties. The weak interfacial adhesion is predominantly due to the hydrophobic surface of talc sheet [3,4], whereas polysaccharides are commonly hydrophilic. Therefore, coupling agents have to be used to promote the interfacial adhesion between the two phases.

Coupling agents have often received considerable attention from researchers in the last few decades due to the ability of these agents to act as a chemical bridge or an interaction link between hydrophilic and hydrophobic substances [5]. Coupling agents such as organotrialkoxysilanes, titanates, zirconates, organic acid-chromium chloride coordination complexes and many others have been studied [6,7]. Recently, ionic liquids were also used as coupling agents in polymeric composite systems. This strategy seems to be exciting due to some fascinating features of the ionic liquids, for instance; non-volatile, environmentally friendly, non-flammable, thermally stable, can be designed, *etc.* [8]. Apart from their unique properties, such materials are on one hand compatible with many organic compounds including biopolymers [9] and on the other hand, they can interact with inorganic compounds including minerals [10].

The use of ionic liquids as coupling agents in polymer composites was first reported by Das *et al.* [11] in 2009. The aim of their research was to study coupling activity of ionic liquids for enhancing the compatibility and dispersibility of multi-walled carbon nanotubes (MWCNTs) with a diene elastomers polymer matrix. They applied five different ionic liquids specifically; 1-allyl-3-methyl imidazolium chloride, 1-ethyl-3-methyl imidazolium thiocyanate, 1-methyl-3-octylimidazolium chloride, 3-(triphenylphosphonic)-1-sulfonic acid tosylate and trihexyl tetradecyl phosphonium decanoate. Among the tested ionic liquids they found that the addition of 1-allyl-3-methyl imidazolium chloride showed a three-fold increase in tensile strength which was achieved with only <3 wt % MWCNT loading for the styrene-butadiene/polybutadiene rubber blend that was used as basic elastomer. The composite can be stretched up to 456% without mechanical failure at this low concentration of MWCNTs.

Additionally, a study employing ionic liquid as a coupling agent in polypropylene-silica composites was reported by Donato *et al.* [12] in 2010. They discovered that the presence of 1-decyl-3-methylimidazolium tetrafluoroborate ionic liquid on the silica surface significantly improved dispersion and prevented compression of the silica particles in the polymer matrix. Very recently, Donato *et al.* [13] used 1-decyl-3-methylimidazolium tetrafluoroborate, 1-triethylene glycol monomethyl ether-3-methylimidazolium tetrafluoroborate and 1-triethylene glycol monomethyl ether-3-methylimidazolium methanesulfonate in the formation of epoxy-silica nanocomposites. It was found that 1-decyl-3-methylimidazolium tetrafluoroborate produced very fine hybrid morphology with well-dispersed silica nanodomains and it has significantly increased the rubbery modulus of nanocomposites. They clarified that the improvement of the properties of the nanocomposites is due to physical crosslinking by the ordered domains of decyl-substituents.

It has been reported that more than 250 ionic liquids have already been successfully produced. One such, 1-n-Butyl-3-methylimidazolium chloride (abbreviated to BmimCl) is ubiquitous and frequently applied as a starting material for production of many room temperature and/or hydrophobic ionic liquids. In addition, recently BmimCl ionic liquid has been studied mostly for the processing of complex macromolecules and polysaccharides. This is due to its compatibility with biopolymeric substances [14]. Besides, BmimCl utilized in this study has been blended with urea at an equal mole ratio to create a BmimCl/urea mixture. It is intended to increase the efficacy of ionic liquid anions for interacting with polysaccharide since each urea molecule has two amide groups, as observed in the early stages of our preceding work [15]. At the same time, the cations of the BmimCl/urea mixture may interact with the edges of the talc particles [16]. Both interactions could lead to a good filler-to-matrix interfacial adhesion and eventually improve the properties of the produced composite films.

Furthermore, to date, the information pertaining to the polysaccharide/mineral composite films that were prepared via a gelation method in the presence of BmimCl/urea mixture has not been reported. Therefore, the present paper aims to study the potential of BmimCl/urea to couple composite films compiled with agarose and talc. The mechanical properties, surface morphologies, thermal properties and chemical interactions of the composite films with and without the addition of BmimCl/urea mixture as a coupling agent were characterized by means of Instron universal testing instrument, scanning electron microscope (SEM), differential scanning calorimetry (DSC), thermogravimetric analysis (TGA), Fourier transform infrared spectroscopy (FTIR) and FT-Raman spectrometry.

## 2. Results and Discussion

### 2.1. Mechanical Testing

**Figure 1:**
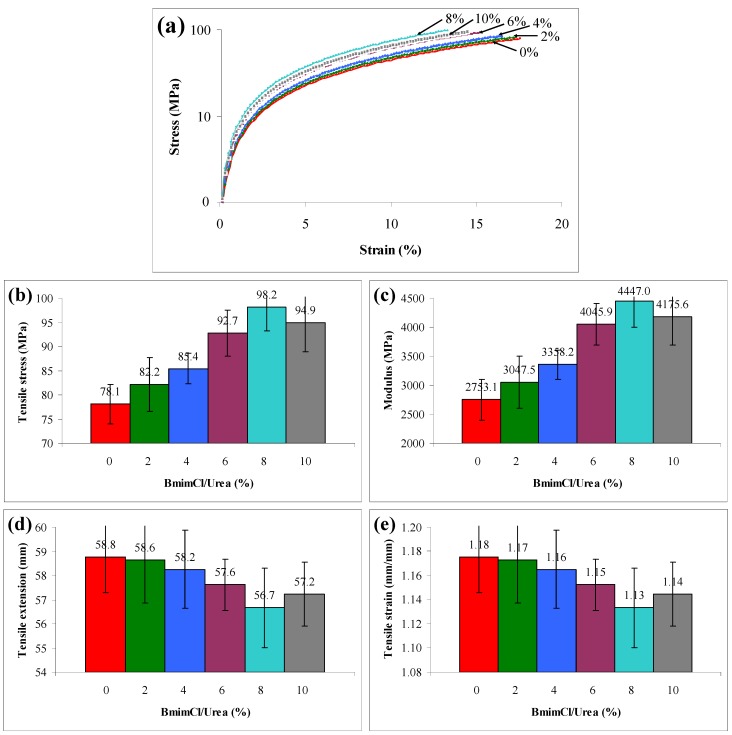
Effects of 1-n-butyl-3-methylimidazolium chloride (BmimCl) content on (**a**) stress-strain behaviour; (**b**) tensile stress; (**c**) modulus; (**d**) tensile extension and (**e**) tensile strain of the agarose/talc composite films.

The stress-strain plot of the agarose/talc composite films with different contents of BmimCl/Urea is shown in Figure 1a. In general, the introduction of the BmimCl/Urea into the composite films increases the stress at break. However, it also causes a further reduction in strain at break in all the composite samples. For example, the addition of 8 wt % BmimCl/Urea in the composite film increased the stress by 26% when compared with the one without BmimCl/Urea. On the contrary it also decreased the strain by 24%. In Figure 1b,c, the tensile stress and modulus at break of the composite film without BmimCl/Urea are lower than those of other composite films. This is commonly due to the poor interfacial adhesion between hydrophilic agarose and hydrophobic talc. This led to the reduction of measured properties since the absorption of the exerted force on the composite films generally takes place at around their interface. On top of that, the composite films that contain BmimCl/Urea showed high tensile stress and modulus compared to the native composite film. The addition of BmimCl/Ureamakes the agarose/talc composite films become stronger and stiffer, hence it could be acting like a coupling agent. Nevertheless, the tensile stress and modulus of the composite increase progressively but with the presence of a higher content of BmimCl/Urea (beyond 8 wt %) a minor decrease was perceived. Therefore, 8 wt % BmimCl/Urea in the composite film was considered the optimum content. The tensile stress at this content increased up to 98.2 MPa and its modulus grew by 4447.0 MPa compared with the composite film without BmimCl/Urea, where the tensile stress and modulus were only 78.1 MPa and 2753.1 MPa, respectively. Conversely, the decrease trend in tensile stress and modulus with higher content of BmimCl/Urea (10 wt %) may be speculated as due to the BmimCl/Urea surpassing its circumscription as a coupling agent in the agarose/talc composite films.

The tensile extension and tensile strain at break for the agarose/talc composite films are shown in Figure 1d,e, respectively. The BmimCl/Urea has slightly reduced the tensile extension and tensile strain of the composite films. These results show that the motion of agarose molecular chains has been restrained by talc particles. The impact is further increased with the presence of BmimCl/Urea, thus achieving the desired strengthening and stiffening effects. However, the composite film produced with 10 wt % BmimCl/Urea exhibited an approximately 1% higher tensile strain and tensile extension at break compared to the one with 8 wt % BmimCl/Urea. This might be due to the addition of the ionic liquids at high loading into polysaccharides which often increases their tensile extension and tensile strain at break resulting from the plasticizing effect of the ionic liquid [15]. In spite of this, the mechanical testing results clearly indicate that the addition of BmimCl/Urea mixture increased the tensile stress and modulus properties of the agarose/talc composite films and it slightly decreased the tensile extension and tensile strain properties.

### 2.2. Morphology Examination

**Figure 2:**
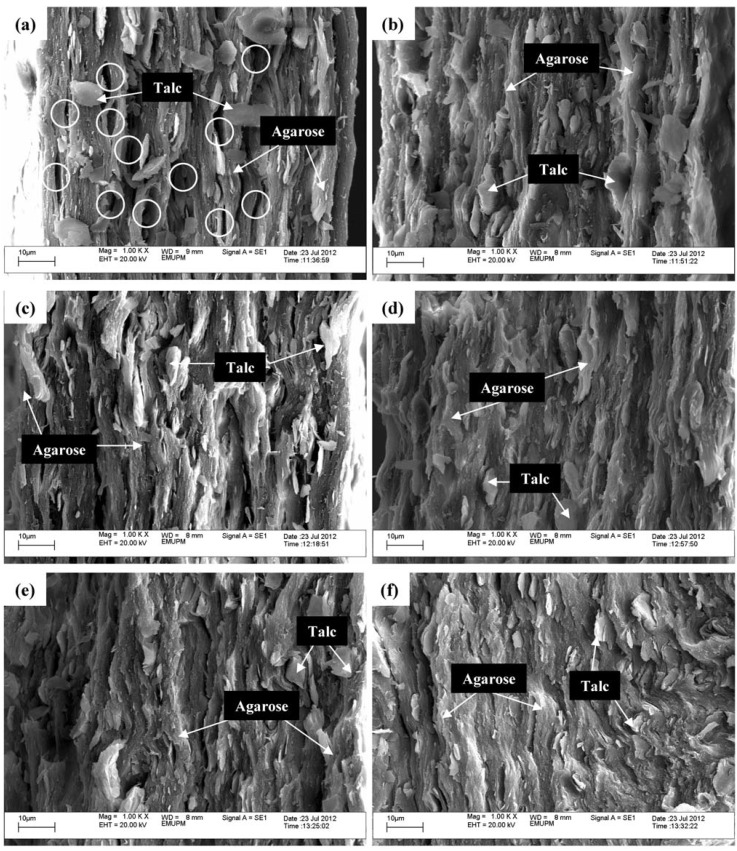
Scanning electron microscope (SEM) micrographs of the fractured surface of the agarose/talc composite films with (**a**) 0 wt %; (**b**) 2 wt %; (**c**) 4 wt %; (**d**) 6 wt %; (**e**) 8 wt % and (**f**) 10 wt % of BmimCl/Urea at magnification of 1000×.

SEM was employed to examine the morphology of the tensile fractured surfaces of the prepared composite films. Figure 2a–f shows the SEM micrographs of the agarose/talc composite films composed of various contents of BmimCl/Urea mixture. In Figure 2a cavities (circled in white) at the fracture surface of the composite can be seen because the particles were pulled out of the matrix after the tensile force had been applied. This is due to poor or insufficient adhesion between fillers and the polymers [17]. This greatly decreases tensile stress and modulus as demonstrated in the mechanical testing results.

Instead, it is apparent in Figure 2b–e with 2 wt % to 8 wt % of BmimCl/Urea, respectively, all the talc particles are embedded in the agarose matrix and there is also no pullout of the talc particles from the composite films. Besides, the wetting of the talc particles surface by agarose is much better than that of the composite film without BmimCl/Urea. Moreover, the diffusion of talc particles is very good and they are dispersed uniformly throughout the agarose matrix. The coupling capability provided by the BmimCl/Urea is the key factor that enhances the dispersion which is thus responsible for the excellent stiffness property. Therefore, the agarose and the talc are totally compatible with one another with the addition of BmimCl/Urea. On the other hand, it can be observed from the SEM micrograph of Figure 2f, that increasing the content of BmimCl/Urea up to 10 wt % indicated the ductile fracture surface characteristic of the composite film in line with its mechanical properties. This is caused by the addition at high loading of BmimCl/Urea which induces a small increase in tensile extension and tensile strain of the composite film as formerly explained in the mechanical testing results. In the morphology observation findings, it was discovered that the addition of the BmimCl/Urea mixture into the agarose/talc composite films also exhibited coupling agent character.

### 2.3. DSC Analysis

**Figure 3:**
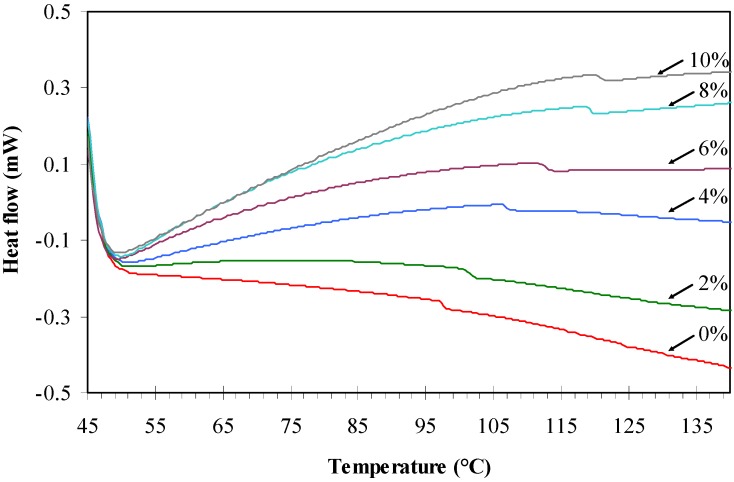
Differential scanning calorimetry (DSC) thermograms of the agarose/talc composite films.

DSC analysis was used to determine the glass transition temperature, *T*_g_ of the agarose/talc composite films. Figure 3 presents the DSC thermograms of the composite films and the trend in variation of the *T*_g_ of the composite films is presented in Table 1. The *T*_g_ value of the composite film without BmimCl/Urea (98.9 °C) is lower in comparison with the composite films containing BmimCl/Urea. The main reason is that the interfacial adhesion between filler and the matrix is already weak before the addition of BmimCl/Urea mixture as previously described in the mechanical testing and morphology examination results. With the addition of BmimCl/Urea an increase in the *T*_g_ of the composite films was observed, this upward trend is attributed to the increase in interaction between the fillers and the polymer matrix [18].

**Table 1:** Glass transition temperature (*T*_g_) of the agarose/talc composite films.

Agarose/talc (wt %)	BmimCl/Urea (wt %)	*T*_g_ (°C)
100	0	98.9
98	2	102.4
96	4	108.5
94	6	113.5
92	8	120.6
90	10	122.1

It also can be seen that the values of *T*_g_ for the composite films increased with increasing content of BmimCl/Urea in the composite films, where 2 wt % and 4 wt % of BmimCl/Urea gave *T*_g_ of 102.4 °C and 108.5 °C, respectively. The 6 wt % of BmimCl/Urea in the composite film showed a higher *T*_g_ of 113.5 °C, followed by 8 wt % with 120.6 °C. The composite film with 10 wt % of BmimCl/Urea demonstrated the *T*_g_ value increased up to 122.1 °C. The increase is due to the formation of a much stronger interaction between the agarose chains and the talc particles. On the other hand, an anomaly was observed for the *T*_g_ of the composite film containing 10 wt % BmimCl/Urea whereby its value was slightly higher than the composite film containing 8 wt % BmimCl/Urea. It seems that the *T*_g_ is not in line with the tensile stress and modulus properties. Nevertheless, this incongruity can be neglected because the disparities in both the *T*_g_ values are not significantly large. The DSC results implied that the BmimCl/Urea mixture could effectively increase the *T*_g_ of the agarose/talc composite films.

### 2.4. TGA Characterization

**Figure 4:**
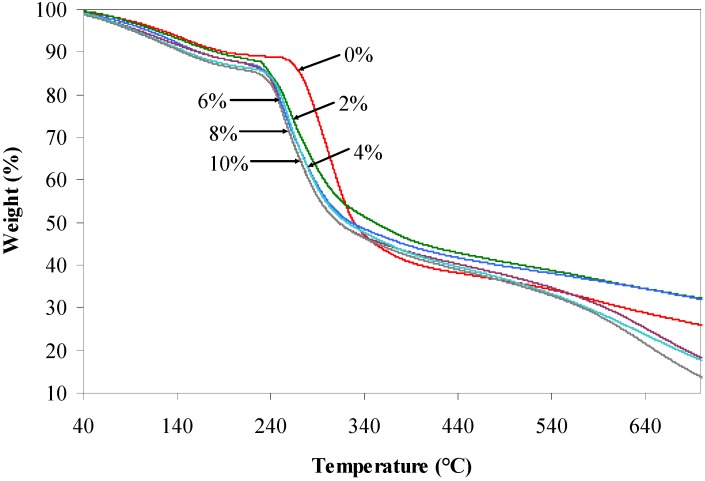
Thermogravimetric analysis (TGA) thermograms of the agarose/talc composite films.

**Figure 5:**
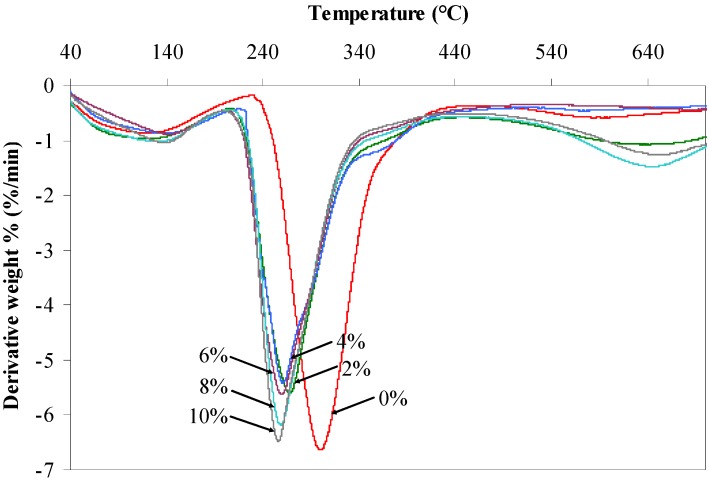
Derivative thermograms (DTG) of the agarose/talc composite films.

TGA characterization was executed to probe the initial and maximum decomposition temperatures of the prepared composite films. TGA thermograms and derivative thermograms (DTG) of the agarose/talc composite films with and without BmimCl/Urea mixture are represented in Figure 4 and Figure 5, respectively.

Meanwhile, Table 2 shows the value of initial decomposition temperature (at 10% weight loss) and the value of maximum decomposition temperature of the composite films. From the analysis, it could be roughly observed that there are two stages of weight loss, associated with the loss of water molecules and their components, respectively. It can be seen that the composite film without BmimCl/Urea loses about 10% of its weight at 192.7 °C. It is known that the talc particles could raise the decomposition temperature of polymer composites [2] but the addition of BmimCl/Urea into agarose/talc composite films significantly decreased the initial decomposition temperature of the composite films as shown in Table 2. This observation is also supported by the DSC results (Figure 3) where the heat flow for the composite films containing more BmimCl/Urea indicated very broad exothermic thermograms immediately after the *T*_g_ which are the result of low thermal decomposition. Thus, the composite film without BmimCl/Urea outperforms the thermal stability of the composite films that contain BmimCl/Urea. Furthermore, similar behaviour was also observed for the maximum decomposition temperature of the composite films with BmimCl/Urea added, that is 267.8 °C, 260.6 °C, 259.7 °C, 258.9 °C and 256.2 °C for the composite films containing BmimCl/Urea of 2 wt %, 4 wt %, 6 wt %, 8 wt % and 10 wt %, respectively. Whilst, the maximum decomposition temperature of the composite film with 0 wt % BmimCl/Urea was 300.1 °C.

**Table 2:** The initial and maximum decomposition temperature obtained from thermogravimetric analysis (TGA) and derivative thermogram (DTG) of the agarose/talc composite films.

Agarose/talc (wt %)	BmimCl/Urea (wt %)	Initial decomposition temperature (°C)	Maximum decomposition temperature (°C)
100	0	192.7	300.1
98	2	182.1	267.8
96	4	163.5	260.6
94	6	163.4	259.7
92	8	150.4	258.9
90	10	146.0	256.2

Although the tensile stress, modulus and *T*_g_ value of the agarose/talc composite films could be greatly increased after BmimCl/Urea is added, their thermal decompositions are inversely proportional to BmimCl/Urea content. This is probably because of the ionic liquid mixture component particularly urea which possesses a lower thermal resistance in comparison to other components of the composite films [19] besides, its decomposition rate being also much faster under a nitrogen atmosphere than under air [20]. Hence, it can be assumed that the thermal decomposition of the agarose/talc composite films is dependent on the thermal stability of their individual components including the ionic liquid mixture component. The BmimCl is blended with urea because it consists of amide groups that are sensitive to hydrogen bonding interaction [21]. Moreover, the double amide groups in each urea molecule enable it to create anion complexes that increase the formation of hydrogen bonding with the agarose [15]. This also could definitely decrease the thermal decomposition temperature of the overall composite films since there is interaction between each of the components formed by BmimCl/Urea. Thereby, the addition of BmimCl/Urea mixture drastically decreases the thermal decomposition of the agarose/talc composite films.

### 2.5. FTIR Analysis

**Figure 6:**
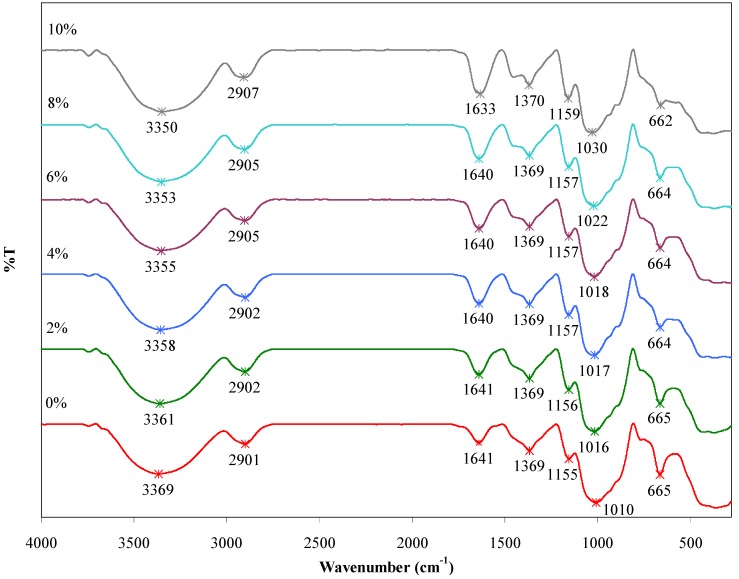
Fourier transform infrared spectroscopy (FTIR) spectra of the agarose/talc composite films with various contents of BmimCl/Urea.

**Figure 7:**
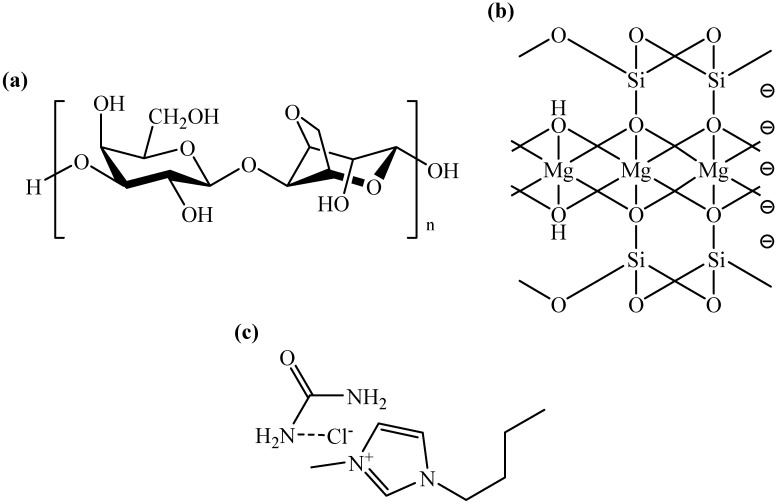
Chemical structures of the (**a**) agarose; (**b**) talc and (**c**) BmimCl/Urea.

FTIR analysis was carried out to identify the functional groups present in the composite films and to ascertain the existence of the interaction between agarose and talc. Figure 6 shows FTIR spectra of the agarose/talc composite films with different contents of BmimCl/Urea. Some important FTIR bands are summarized in Table 3. The FTIR spectra of the composite films display broad bands with strong intensity in the region of 3369 to 3350 cm^−1^ that are responsible for the O–H bond stretching vibration of the alcohol group. The bands with medium intensity at around 2907 to 2901 cm^−1^ are associated with C–H bond stretching of the alkane group. Adsorbed water is ascribed to the characteristic bending vibration at bands ranging from 1641 to 1633 cm^−1^. The bands with medium intensity at around 1370 to 1369 cm^−1^ are due to CH_3_ bond bending of the alkane group. The bands from 1159 to 1155 cm^−1^ with medium intensity could be ascribed to the C–O–C bond stretching of the ether group of the agarose matrix [14]. The presence of talc in the composite films was proven by the bands in the range of 1030 and 662 cm^−1^. The Si–O–Si bond stretching of the siloxane group is indicated by the bands at around 1030 to 1010 cm^−1^ with strong intensity [22], whilst the bands with medium intensity at 665 to 662 cm^−1^ are designated to Si–O–Mg stretching vibrations [23]. The chemical structures of the agarose, talc and BmimCl/Urea are shown in Figure 7a–c, respectively.

**Table 3:** FTIR bands of the agarose/talc composite films.

BmimCl/Urea (wt %)	Wavenumber (cm^−1^)					
	O–H stretching	C–H stretching	CH_3_ bending	C–O–C stretching	Si–O–Si stretching	Si–O–Mg stretching
0	3369	2901	1369	1155	1010	665
2	3361	2902	1369	1156	1016	665
4	3358	2902	1369	1157	1017	664
6	3355	2905	1369	1157	1018	664
8	3353	2905	1369	1157	1022	664
10	3350	2907	1370	1159	1030	662

**Figure 8:**
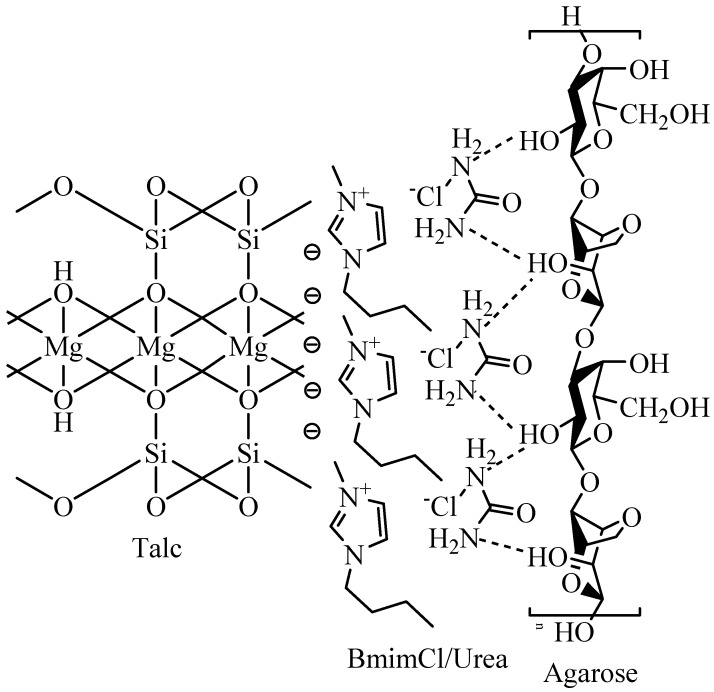
Proposed interactional model for the talc–BmimCl/Urea–agarose in the composite films.

On the other hand, FTIR spectra of the agarose/talc composite films (Figure 6) show almost no difference in terms of the pattern. Yet, in Table 3 for the composite films containing BmimCl/Urea, there are two major band shifts, specifically associated with O–H and Si–O–Si stretching. The O–H stretching of the alcohol group of the agarose matrix was found to shift to a lower wavenumber with downshifted maximum at 19 cm^−1^. This showed the existence of the interaction between agarose with talc particles due to the addition of BmimCl/Urea [14,24]. In contrast, the Si–O–Si stretching of the siloxane group of the talc particles also shifted but significantly towards higher wavenumbers, with a maximum shift up to 20 cm^−1^. The observed shifts were caused by the formation of an interaction between fillers with the polymer matrix [25]. Regarding the interaction, the addition of BmimCl/Urea into the composite films actually results in two different interactions; those are BmimCl/Urea–agarose interaction and talc–BmimCl/Urea interaction. The BmimCl/Urea–agarose interaction is expected between the amide groups in the BmimCl/Urea with the hydroxyl groups of the agarose via hydrogen bonding [15]. The talc–BmimCl/Urea interaction was considered between the negatively charged edges of the siloxane group of the talc particles with the cations moiety that is present in the BmimCl/Urea mixture through van der Waals force [16,26]. Accordingly, an interactional model was proposed as presented in Figure 8. In addition, the BmimCl/Urea could strongly hold everything together (talc–BmimCl/Urea–agarose) via ionic bonding due to its ionic nature. As a result, the talc was attached more strongly to the agarose matrix. These interactions could improve the adhesion of the two-phase interface, consequently the transfer of stress or energy from the matrix to the fillers surfaces is increased. This is in agreement with the significant enhancement in the mechanical, morphological and thermal (*T*_g_) properties of the final composite films. Based on the FTIR results, the interactions of agarose matrix molecular chains and the talc particles exist with the addition of BmimCl/Urea mixture.

### 2.6. FT-Raman Study

**Figure 9:**
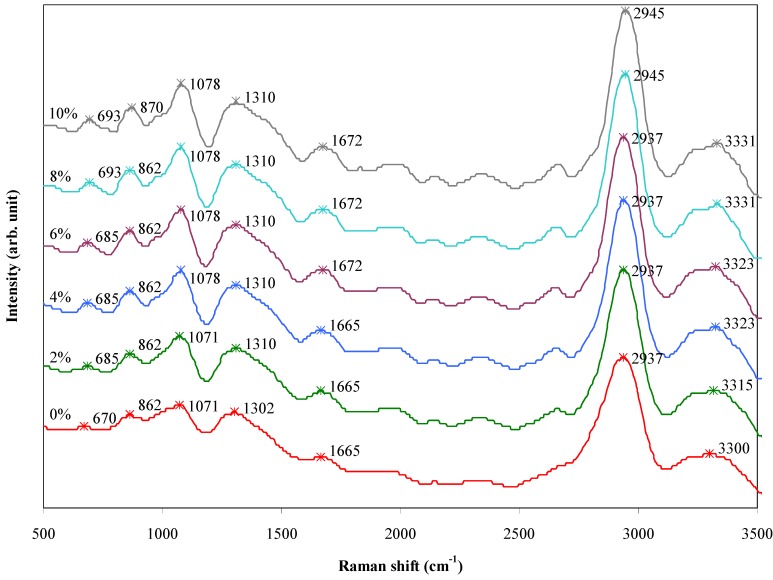
FT-Raman spectra of the agarose/talc composite films containing different amount of BmimCl/Urea.

FT-Raman study was conducted as a complementary method in order to ensure the presence of the interaction between the agarose matrix and talc particles in the prepared composite films. Figure 9 shows the FT-Raman spectra of the agarose/talc composite films with different contents of BmimCl/Urea. The assignment and functional group of the characteristic FT-Raman bands of the composite films are summarized in Table 4. The bands with low intensity at around 670 to 693 cm^−1^ can be assigned to the SiO_4_ stretching vibration of the silica group [27], which is consistent with the existence of talc in each composite film. The C–H bond bending vibration of the alkane group revealed medium intensity bands at around 862 to 870 cm^−1^ [28]. The medium intensity bands at *circa* 1071 to 1078 cm^−1^ are believed to be a feature of C–O stretching of the alcohol group. The existence of CH_3_ bending was validated in the bands range of 1302 to 1310 cm^−1^ with medium intensity. The bands at around 1665 to 1672 cm^−1^ were attributed to the presence of tightly bound water. The C–H stretching of the alkane group was verified at 2937 to 2945 cm^−1^ with strong intensity. The broad bands around 3300 to 3331 cm^−1^ with medium intensity are confirmed as O–H bond stretching of the alcohol group [29].

**Table 4:** The assignment and functional groups of FT-Raman bands of the agarose/talc composite films.

Raman shift (cm^−1^)	Assignment	Functional group
670–693	SiO_4_ stretching	Silica
862–870	C–H bending	Alkane
1071–1078	C–O stretching	Alcohol
1302–1310	CH_3_ bending	Alkane
2937–2945	C–H stretching	Alkane
3300–3331	O–H stretching	Alcohol

From the FT-Raman spectra, it can be seen that the majority of the bands display insignificant shifts of only 7 to 8 cm^−1^. On the contrary, the SiO_4_ stretching of the silica group and the O–H stretching of the alcohol group exhibit substantial blue shifts (increase) in the wavenumber of 15 to 31 cm^−1^ with increase of the BmimCl/Urea content in the agarose/talc composite films. The large hypsochromic shift has been attributed to the presence of the interactions that improve interfacial adhesion between the polymer matrix and the fillers [11]. Hence, it is interesting to mention that the FT-Raman results also indicated the presence of interaction between agarose and talc in the composite films. Thus, the utilization of BmimCl/Urea mixture as a coupling agent to improve the interfacial adhesion between the hydrophobic talc particles and the hydrophilic agarose matrix is not impossible. Nonetheless, BmimCl/Urea is not considered to be a chemical coupling agent since no primary bonding (covalent bond) was formed between agarose and talc, because no new distinct bands can be seen in both the FTIR and FT-Raman spectra of the composite films. The effect of secondary bondings (hydrogen bonding and van der Waals forces) significantly causes a hypsochromic shift of the bands of the silica and alcohol groups after the addition of BmimCl/Urea mixture and therefore, it is conveniently referred to as a physical coupling agent for the agarose/talc composite films.

## 3. Experimental Section

### 3.1. Materials

The ionic liquid used 1-n-butyl-3-methylimidazolium chloride (BmimCl) (≥95%) was procured from Sigma Aldrich. Urea (purist grade) was also purchased from Sigma Aldrich. The biopolymeric matrix used was agarose (analytical grade) obtained from Merck. The filler composite was talc powder (10 microns) acquired from Sigma Aldrich. All the chemicals were used as received without further treatment. Distilled water was employed as a medium for the preparation of the composite films.

### 3.2. Preparation of BmimCl/Urea Mixture and Agarose/Talc Composite Films

The mixture of BmimCl/Urea was made by blending BmimCl with urea in a beaker at 1:1 mole ratio. The mixture was then heated to 100 °C and stirred vigorously until a transparent homogeneous liquid was obtained. The agarose/talc composite films were prepared by placing 0.04 g of BmimCl/Urea and 1.6 g of agarose into a 100 mL beaker followed by 0.4 g of talc and 56 g of distilled water. They were then heated at 95 °C and stirred at 1500 rpm by using a magnetic stirring apparatus until all of the agarose was dissolved. The solution was immediately poured into a 14 cm internal diameter glass Petri dish and allowed to cool to room temperature (25 °C) for the gelation process. Later the gel was slowly dried at a temperature of 40 °C for 48 h to obtain a freestanding composite film containing 2 wt % of BmimCl/Urea. The dish was exposed to ambient air to ease the film peel-off from the surface. Prior to characterizations, the resultant composite films were dried in an oven at 80 °C for at least 24 h. The weight ratio of agarose and talc was fixed at 4:1, while the content of BmimCl/Urea was varied from 2 to 10 wt % relative to the overall weight of the composite films. A composite film containing only agarose and talc (0 wt % BmimCl/Urea) was also prepared for comparison purpose.

### 3.3. Characterization

Mechanical testing of the prepared composite films was performed with an Instron Universal Testing Instrument (model 5566) equipped with a 1 kN load cell according to ASTM D882-91 method [30] at room temperature with a relative humidity of 56% (±5%). The composite film samples were cut into a rectangular shape with a length and width of 75 mm and 10 mm, respectively. The initial grid separation was set to 50 mm and the crosshead speed was 5 mm min^−1^. The readings were taken from seven samples for each BmimCl/Urea contents.

Morphology examination of the composite film samples was done by using LEO (model 1455) VPSEM. The samples were cross-sectioned by making a tensile fracture at room temperature. The fractured surfaces of the samples were mounted on aluminium stubs and sputter-coated with a thin layer of gold to avoid electrostatic charge during examination. The samples were observed at a magnification of 1000× with accelerating voltages of 20.0 kV.

DSC analysis of the prepared composite films was operated on a Mettler Toledo 822^e^ instrument. All samples were tightly sealed in aluminium pans and were subjected to the following procedure; the samples were first heated to 100 °C and held at this temperature for 15 min to eliminate thermal history. After that, the samples were slowly cooled to 45 °C at a rate of 10 °C min^−1^ before they were subsequently heated to 140 °C at the same heating rate.

TGA of the composite film samples was executed by using the Perkin Elmer TGA7 instrument. The samples were analyzed with a heating rate of 10 °C min^−1^ and the temperature ranged from 37 to 700 °C under nitrogen atmosphere at a flow rate of 50 mL min^−1^.

FTIR analysis of the prepared composite films was carried out by means of Perkin Elmer Spectrum 100 Series. The FTIR spectra were obtained by using a universal attenuated total reflectance (UATR) equipped with a ZnSe-diamond composite crystal accessory. Each spectrum was 16 scans, in the frequency range of 4000 to 280 cm^−1^ and resolution of 4 cm^−1^.

FT-Raman study of the composite films samples was conducted using a Bruker RFS 100/S FT-Raman spectrometer. The incident laser excitation was provided by Nd:YAG laser source operating at wavelength of 1064 nm and the power of 450 mW with InGaAs detector in the frequency range from 500 to 3500 cm^−1^. Each sample was scanned 5 times at a resolution of 50 cm^−1^. All samples were characterized under identical experimental conditions.

## 4. Conclusions

From this study, the optimum content of BmimCl/Urea in the agarose/talc composite film with highest tensile stress and modulus properties was found to be 8 wt %. SEM micrographs for the composite films containing BmimCl/Urea demonstrated that the talc particles are embedded in the agarose matrix and there are no pullouts. The glass transition temperature, *T*_g_ values of the composite films increased with the addition of BmimCl/Urea, however the thermal decomposition temperature decreased drastically. The significant shifts of the bands of FTIR and FT-Raman spectra with the addition of BmimCl/Urea imply the existence of interaction, which serves to improve the interfacial adhesion of the composite films. It is inferred that a BmimCl/Urea mixture can be utilized as a physical coupling agent between agarose and talc in a composite films system.

## References

[1] Leong Y.W., Abu Bakar M.B., Ishak Z.A.M., Ariffin A., Pukanszky B. (2004). Comparison of the mechanical properties and interfacial interactions between talc, kaolin, and calcium carbonate filled polypropylene composites. J. Appl. Polym. Sci..

[2] Zhou Y., Rangari V., Mahfuz H., Jeelani S., Mallick P.K. (2005). Experimental study on thermal and mechanical behavior of polypropylene, talc/polypropylene and polypropylene/clay nanocomposites. Mater. Sci. Eng. A.

[3] Wang J., Somasundaran P. (2006). Mechanisms of ethyl(hydroxyethyl) cellulose–solid interaction: Influence of hydrophobic modification. J. Colloid Interface Sci..

[4] Flaris V., Xanthos M. (2005). Talc. Functional Fillers for Plastics.

[5] Kord B. (2011). Evaluation on the effect of wood flour and coupling agent content on the hygroscopic thickness swelling rate of polypropylene composites. BioResources.

[6] Sarang S., Misra R.D.K. (2004). Strain rate sensitive behavior of wollastonite-reinforced ethylene–propylene copolymer composites. Mater. Sci. Eng. A.

[7] González J., Albano C., Ichazo M., Diaz B. (2002). Effects of coupling agents on mechanical and morphological behavior of the PP/HDPE blend with two different CaCO_3_. Eur. Polym. J..

[8] Shamsuri A.A., Abdullah D.K. (2010). Protonation and Complexation Approaches for Production of Protic Eutectic Ionic Liquids. J. Phys. Sci..

[9] Shamsuri A.A., Abdullah D.K. (2012). A Preliminary Study of Oxidation of Lignin from Rubber Wood to Vanillin in Ionic Liquid Medium. Oxid. Commun..

[10] Łuczak J., Joskowska M., Hupka J. (2008). Imidazolium ionic liquids in mineral processing. Physicochem. Probl. Miner. Process..

[11] Das A., Stöckelhuber K.W., Jurk R., Fritzsche J., Klüppel M., Heinrich G. (2009). Coupling activity of ionic liquids between diene elastomers and multi-walled carbon nanotubes. Carbon.

[12] Donato R.K., Benvegnú M.A., Furlan L.G., Mauler R.S., Schrekker H.S. (2010). Imidazolium salts as liquid coupling agents for the preparation of polypropylene-silica composites. J. Appl. Polym. Sci..

[13] Donato R.K., Matějka L., Schrekker H.S., Pleštil J., Jigounov A., Brus J., Šlouf M. (2011). The multifunctional role of ionic liquids in the formation of epoxy-silica nanocomposites. J. Mater. Chem..

[14] Shamsuri A.A., Abdullah D.K., Daik R. (2012). Fabrication of agar/biopolymer blend aerogels in ionic liquid and co-solvent mixture. Cell. Chem. Technol..

[15] Shamsuri A.A., Daik R. (2012). Plasticizing effect of choline chloride/urea eutectic-based ionic liquid on physicochemical properties of agarose films. BioResources.

[16] Burdukova E., Becker M., Bradshaw D.J., Laskowski J.S. (2007). Presence of negative charge on the basal planes of New York talc. J. Colloid Interface Sci..

[17] Radford D.W., Grabher A., Bridge J. (2009). Inorganic polymer matrix composite strength related to interface condition. Materials.

[18] Song Z., Hou X., Zhang L., Wu S. (2011). Enhancing crystallinity and orientation by hot-stretching to improve the mechanical properties of electrospun partially aligned polyacrylonitrile (PAN) nanocomposites. Materials.

[19] Schaber P.M., Colson J., Higgins S., Thielen D., Anspach B., Brauer J. (2004). Thermal decomposition (pyrolysis) of urea in an open reaction vessel. Thermochim. Acta.

[20] Fang H.L., DaCosta H.F. Thermolysis characterization of urea-SCR. Proceeding of the 8th Annual Conference of the Engine-Efficiency and Emissions Research (DEER).

[21] Roohpour N., Wasikiewicz J., Moshaverinia A., Paul D., Rehman I., Vadgama P. (2009). Isopropyl myristate-modified polyether-urethane coatings as protective barriers for implantable medical devices. Materials.

[22] Sprynskyy M., Gadzała-Kopciuch R., Nowak K., Buszewski B. (2012). Removal of zearalenone toxin from synthetics gastric and body fluids using talc and diatomite: A batch kinetic study. Colloids Surf. B Biointerfaces.

[23] Yang H., Du C., Hu Y., Jin S., Yang W., Tang A., Avvakumov E.G. (2006). Preparation of porous material from talc by mechanochemical treatment and subsequent leaching. Appl. Clay Sci..

[24] Wang Z., Latonen R., Kvarnström C., Ivaska A., Niu L. (2010). Preparation of multi-walled carbon nanotube/amino-terminated ionic liquid arrays and their electrocatalysis towards oxygen reduction. Materials.

[25] Magniez K., Chaffraix T., Fox B. (2011). Toughening of a carbon-fibre composite using electrospun poly(hydroxyether of bisphenol a) nanofibrous membranes through inverse phase separation and inter-domain etherification. Materials.

[26] Lu Z. (2003). Chemical Coupling in Wood-Polymer Composite. Ph.D. Thesis.

[27] de Veij M., Vandenabeele P., de Beer T., Remon J.P., Moens L. (2009). Reference database of Raman spectra of pharmaceutical excipients. J. Raman Spectros..

[28] Pereira L., Sousa A., Coelho H., Amado A.M., Ribeiro-Claro P.J.A. (2003). Use of FTIR, FT-Raman and ^13^C-NMR spectroscopy for identification of some seaweed phycocolloids. Biomol. Eng..

[29] Kizil R., Irudayaraj J. (2006). Discrimination of irradiated starch gels using FT-Raman spectroscopy and chemometrics. J. Agric. Food Chem..

[30] American Society for Testing and Materials (1992). ASTM D882-91: Standard Test Method for Tensile Properties of Thin Plastic Sheeting.

